# Enhanced Stretch Formability of AZ31 Magnesium Alloy Thin Sheet by Induced Precompression and Sequent Annealing

**DOI:** 10.3390/ma11081401

**Published:** 2018-08-10

**Authors:** Lifei Wang, Bo Song, Zhengyong Zhang, Hua Zhang, Tingzhuang Han, Xiaoqing Cao, Hongxia Wang, Weili Cheng

**Affiliations:** 1 Shanxi Key Laboratory of Advanced Magnesium-Based Materials, Taiyuan 030024, China; 2 Faculty of Materials and Energy, Southwest University, Chongqing 400715, China; bosong@swu.edu.cn; 3 College of Materials Science and Engineering, Taiyuan University of Technology, Taiyuan 030024, China; zhangzhengyong@126.com (Z.Z.); zhanghua2009@126.com (H.Z.); caoxiaoqing@tyut.edu.cn (X.C.); wanghxia1217@163.com (H.W.); 4 College of Materials Science and Engineering, Taiyuan University of Science and Technology, Taiyuan 030024, China; htzhhbhs@163.com

**Keywords:** precompression, annealing, grain growth, texture, stretch formability

## Abstract

In this study, precompression deformation with a strain level of 5.38% along the transverse direction (TD) at room temperature was conducted on a AZ31 magnesium alloy thin sheet with thickness of 1mm. Then subsequent annealing treatment was carried out at various temperatures (200, 300, 400, and 500 °C) to induce static recrystallization (SRX) and grain growth. The stretch formability was also investigated using the hemispherical test. The results showed that the twinning texture induced by the precompression process was nearly inherited by recrystallized grains after annealing process. Grains grew up and the size increased with the increase of annealing temperature. The largest grain size was obtained when annealing at 400 °C. The mechanical properties including strength and ductility decreased due to the development of coarse grains, however, the stretch formability was enhanced significantly. Indeed, the IE-value increased from 2.83 mm in the as-received Mg alloy sheet to 5.78 mm in the precompressed and 400 °C annealed specimens, leading to an improvement of 104%. This was ascribed to the rotated grain orientation and higher activity of (10–12) twins in coarse grains.

## 1. Introduction

Due to their low density, high specific strength, and good damping properties, Mg alloys have been attractive for use in electronic, automotive, aerospace, and biomedical applications [1,2]. However, there are limited slipping systems because of their hexagonal close packed (HCP) structure. The basal plane provides only two independent slipping systems which cannot fit the Von–Mises criterion [3]. Thus, Mg alloys usually exhibit a poor formability at room temperature. Therefore, it is a subject of interest to enhance the stretch formability of Mg alloys using various techniques; weakening basal texture has proved to be an effective way.

Thus far, introducing alloy elements and imposing shear deformation to weaken the basal texture are the mostly widely used methods. Chino et al. [4] added 0.2% Ce into pure Mg then hot rolled it at 673 K. The results indicated that the basal texture of the Mg–0.2Ce alloy was weakened, and thereby the stretch formability (IE-value) was enhanced by 22.5% at room temperature compared to pure Mg. On the other hand, introducing shear deformation to promote grain rotation is another effective method in formability improvement. Huang et al. indicated that hot rolling on AZ31 [5], AZ61 [6], and AZ80 [7] alloys above 773 K would lead to grain rotation from the normal direction (ND) to rolling direction (RD) which resulted in basal texture weakening.

However, above works are mainly focused on the evolution of macro-texture. It is well-known that the microstructure also plays an important role on the evolution of texture, especially tension twins and recrystallization grains. When tension twins occur, the basal plane will rotate about 86.3°. Moreover, grain nucleation and growth during the recrystallization process can also result in basal texture weakening which has a significant effect on subsequent deformation. Xin et al. [8] indicated that AZ31 Mg alloy plates could be rolled 50% in a single pass at 300 °C after introducing {10–12} tension twins by pre-rolling along transverse direction (TD). In contrast, cracks occurred when rolling reduction reached 30% for initial plates. Recently, it has been reported that recrystallization can be promoted by {10–12} tension twins. Basu et al. [9] also showed the dynamic recrystallization (DRX) process could be promoted by initial twins on Mg-1 wt. % Gd and Mg-1 wt. % Ce alloys. Therefore, it can be concluded that twinning, recrystallization, and indeed twinning-induced recrystallization are the influential factors in improving the plasticity of Mg alloys. However, twinning can be activated only when tension and compression loads are perpendicular and parallel to the basal plane, respectively [10]. Moreover, a buckling failure during pre-twins is highly likely in the thin sheet which prevents the occurrence of tension twins. In this study, a special die was developed to avoid the problem and thus, the improvement of stretch formability of the AZ31 alloy sheet was aimed to be achieved through recrystallization grain growth.

## 2. Experimental Procedure

**Figure 1:**
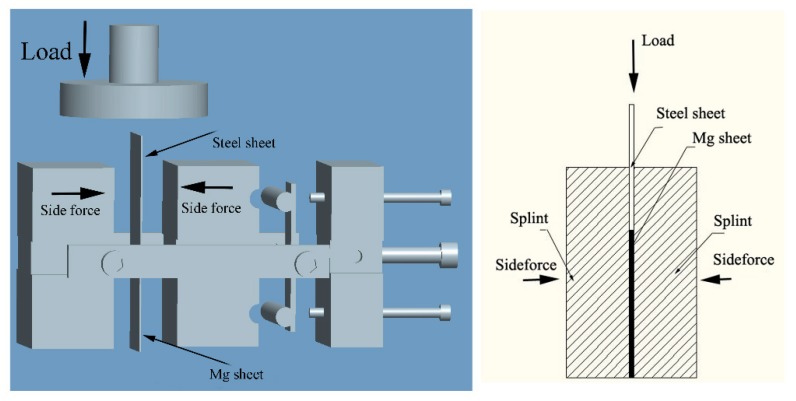
Diagrams of magnesium sheet compression die.

Commercially rolled AZ31 (Mg-3 wt % Al-1 wt % Zn) Mg alloy sheets with thickness of 1mm were used in this experiment. Rectangular specimens were cut from the initial sheet with dimensions of 50 mm (length) and 50 mm (width) along the rolling direction (RD). Then, precompression deformation was conducted along transverse direction (TD) at strain level of 5.38%. Then subsequent annealing treatment was performed at 200 °C, 300 °C, 400 °C, and 500 °C for 2 h. The conditions investigated for the as-received and precompressed AZ31 alloy sheets which anneal at different temperatures can be defined as as-received, PCA200, PCA300, PCA400, and PCA500. In order to make clear the twinning effect on the microstructure evolution, a short time annealing of approximately 10, 20, 40, 60, 80, 100, and 120 s on precompressed samples were also conducted at 400 °C. After keeping the specimens for various times, they were taken out from the furnace quakily and quenched in water. Similar procedures were also carried out on as-received sheets for 30, 60, 90, and 120 s. Precompression specimens without annealing can be likened to PC sheets at the same time. Since the bending-buckling failure is highly likely in thin sheets a special die was developed, as shown in Figure 1. Firstly, the Mg sheet was clamped by two splints. The side force was provided by pressing a spring steel plate through the screws. Grease was used as a lubricant between the Mg sheet as well as two splints to reduce friction. Besides, a high-strength steel sheet (Cr12) with the same thickness was placed on the top of Mg alloy sheet specimens as a pressing plate. Since the strength of steel is much higher than that of Mg alloy sheet, the deformation of the pressing plate can be neglected.

Dog-bone tensile specimens were machined from the pre-strain and annealed Mg sheets with gauge length of 24 mm × 3 mm along RD, 45°, and TD. Tensile tests were performed using DNS2000 electronic universal testing machine at room temperature. The strain rate was set as 10^−3^ s^−1^. The strain hardening exponent (n-value) was determined from the uniform plastic deformation region of the tensile stress–strain curve. Strain along thickness and width was measured to obtain the Lankford value (r-value) using specimens deformed to the strain of 12%. To secure statistically valid outcomes from the testing, each test was repeated three times. Rectangular blanks with a size of 45 mm × 45 mm machined from the precompression and annealed AZ31 alloy specimens were used for Erichsen testing. The Erichsen test was carried out using a hemispherical punch with a diameter of 20 mm at room temperature. The punch speed was set at 10 mm/min and the blank-holder force was 10 KN. A graphite grease was used as a lubricant on the central part of the specimens.

The microstructure was examined by a standard optical microscopy (Leica 2500M, Wetzlar, Germany) (0002). Pole figures were measured by electron backscatter diffraction (EBSD) analysis using a SEM (GEOL GSM-7800F FEG SEM, Chongqing, China) equipped with an HKL Chanel 5 System.

## 3. Results and Discussion

### 3.1. Microstructure and Texture

**Figure 2:**
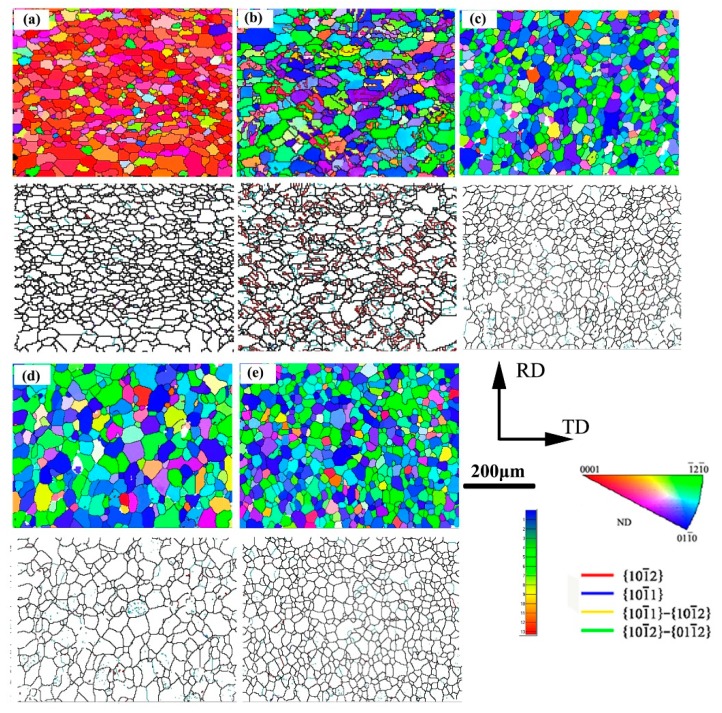
IPF maps and grain boundary distributions of (**a**) as-received, (**b**) PCA200, (**c**) PCA300, (**d**) PCA400, and (**e**) PCA500.

Figure 2 shows IPF maps and grain boundary distributions of the precompression and annealed samples. It can be seen that most of grains lay on the basal plane on the as-received sample and express a red color. After precompression and annealing processes, the basal plane is rotated and expresses a blue color. When annealing at 200 °C, twinning lamellas remain in the microstructure. From grain boundary distribution map (Figure 2b), they can be classified as {10–12} tension twinning structures. Guo et al. [11] indicated that low temperature heat treatment resulted in dislocation annihilations, however, twins can remain. It can also be observed that gains grew up and reached a saturation level (around 400 °C).

The mean grain sizes of as-received, PCA300, PCA400, and PCA500 samples were about 9.6 μm, 21.3 μm, 32.9 μm, and 28.1 μm, respectively. This is mainly due to the occurrence of static recrystallization (SRX). As is well-known, grain boundaries, shear bands, deformation twins, and second phase particles are four preferential nucleation sites for recrystallization in deformed Mg alloys. In the case of twins, different types have different contributions to the recrystallization nucleation and subsequent grain growth. Martin et al. [12] reported that recrystallization within {10–12} extension twins rarely occurred since the matrix within these twins is unfavorably oriented for basal slip. Indeed, the stored energy required for recrystallization could not be accumulated without sufficient basal slips. Guan et al. [13] found that no recrystallization behaviors took place even at the intersection of two large tension twins. In contrast, {10–11} compression twins and {1011}-{1012} double twins always acted as nucleation sites during recrystallization due to the high local strain. However, only {10–12} tension twins can be activated after precompression on AZ31 Mg alloy sheets along TD in this experiment (as shown in Figure 2b). Therefore, the grain nucleation within tension twins is quite limited. Recently, Levinson et al. [14] conducted in situ EBSD on prestrained AZ31 Mg alloys which contained a large amount of {10–12} tensile twins. The results showed that the thermal activation strain induced grain boundary migration (SIBM) happened during annealing. As well known, SIBM is one of the classical nucleation mechanisms during recrystallization of Mg alloys. Jin et al. [15] reported that the driving force for grain growth induced by SIBM is the difference in strain dislocation densities between the relatively strain-free new grains and the strain hardened parent matrix. DRXed grains are first nucleated in twinning deformed regions then grow into the parent matrix by SIBM. The boundaries move faster towards highly deformed regions. As deformation continues the dislocation density increases, which reduces the driving force for grain growth and eventually, the grain boundary motion ceases. Thus, grain growth occurs after precompression and annealing.

**Figure 3:**
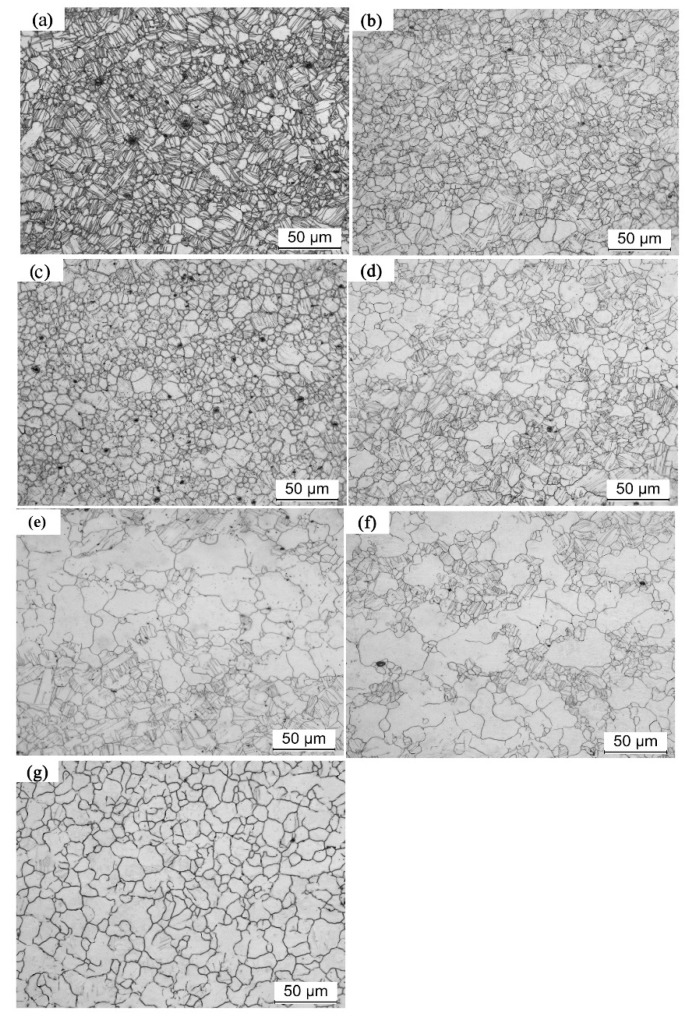
Microstructure evolution of PC400 Mg alloy sample at different annealing times: (**a**) 10 s, (**b**) 20 s, (**c**) 40 s, (**d**) 60 s, (**e**) 80 s, (**f**) 100 s, and (**g**) 120 s.

**Figure 4:**
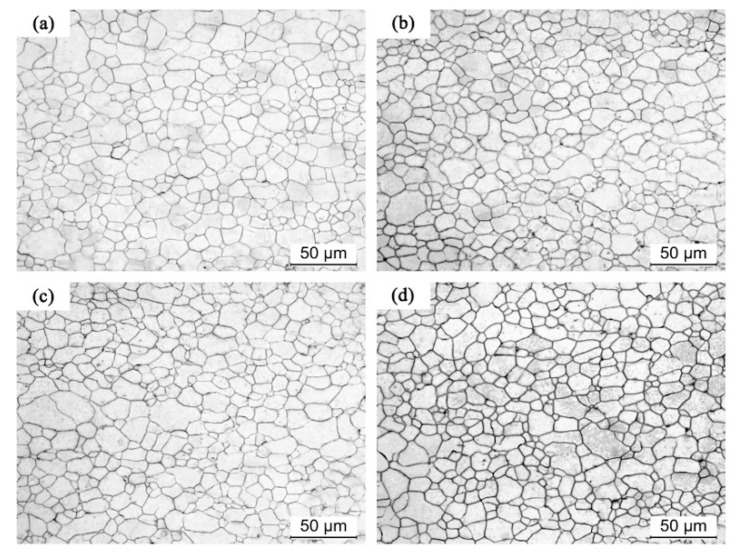
Microstructure evolution of as-received sample at different times: (**a**) 30 s, (**b**) 60 s, (**c**) 90 s, and (**d**) 120 s.

**Figure 5:**
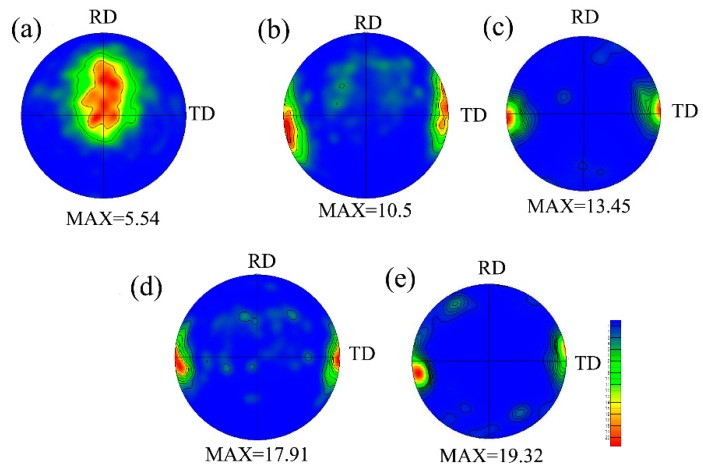
(0002) pole figures of (**a**) as-received, (**b**) PCA200, (**c**) PCA 300, (**d**) PCA 400, and (**e**) PCA 500.

Figure 3 shows the microstructural evolution of precompressed AZ31 Mg specimen annealing at 400 °C (PC400) with various short times (10, 20, 40, 60, 80, 100, and 120 s). For comparison purposes, the microstructure of as-received samples with the same heat treatment are shown in Figure 4. For sample PC400, the recrystallization behavior almost completed in 120 s, as shown in Figure 3. Moreover, within 10~40 s of annealing, the volume fraction of twinning lamellas slightly changed. As annealing time increases, the fraction of {10–12} tension twins decreased obviously. It should be noted that, recrystallized grains and twinning lamellas were simultaneously distributed in the microstructure between 60 s and 100 s. Eventually, nearly all twins disappeared after 120 s annealing at 400 °C, and continuation of annealing up to 2 h resulted in a constant growth of the recrystallized grains. In contrast, as-received Mg alloy specimens showed a rather stable microstructure during annealing at the same condition. Thus, recrystallized grains initiated along locally heavy strained grain boundary segments in a short annealing time. With the annealing time increasing, those grains grew into the deformed parent grains and tension twins, thus recrystallization occurred by subgrain boundary migration. Basically, the tension twins are consumed by their adjacent recrystallized grain. It means that the nucleation took place on the heavily strained grain boundaries in precompressed Mg sheets in a short annealing time. As the annealing time increases, {10–12} tension twins are consumed by adjacent recrystallized grains which contributes to the grain growth. Eventually, the grain boundaries spread faster and bigger grains were obtained at higher annealing temperatures, as shown in Figure 3. In addition, previous research [16] reported that there was a critical store energy for nucleation and grain growth during static recrystallization in Mg alloys. Indeed, the grain growth rate is higher than that of the nucleation and vice versa below the critical store energy. This explains why the biggest grain size was achieved at 400 °C. Accordingly, this annealing temperature may be a critical temperature in precompressed Mg alloy sheets which need to be investigated more in the future.

(0002) pole figures of the investigated samples are shown in Figure 5. The as-received sample features a typical basal texture with an intensity of 5.54. After precompression, the basal plane rotated by 86.3° toward TD, confirming the activation of {10–12} tensile twins. The precompression induced texture remained almost unchanged even after annealing in the range of 200–500 °C. In general, most of the basal plane rotates towards the initial basal plane after static recrystallization (SRX). Conversely, in this study, the twinning texture was reserved after annealing. This is mainly related to the strain-induced boundary migration (SIBM) during recrystallization. Due to SIBM, the recrystallized grains (green or blue grains shown in Figure 5) exhibit similar orientations to those of {10–12} twins. Besides, the limited stored energy was not adequate for grain nucleation, while it was enough for grain growth. The disappearance of {10–12} tensile twins is mainly related to the consuming by the growth of recrystallized grains. During the grain growth, the consumption of the matrix by twins contributes to stabilization of the texture component around TD. Moreover, as the annealing temperature increased, the thermal activation energy increased and thereby, the {10–12} tension twins were consumed faster, and larger texture intensity was obtained. Xin et al. [17] also reported the basal pole around TD was well kept when precompressed along ND for 5.5% then recompression along TD for 2.8% and annealed at 250 °C for 1 h. The research also traced the single parent grain and found that the orientation of recrystallized grains followed those of their neighboring parent grains that expressed a similar result.

**Figure 6:**
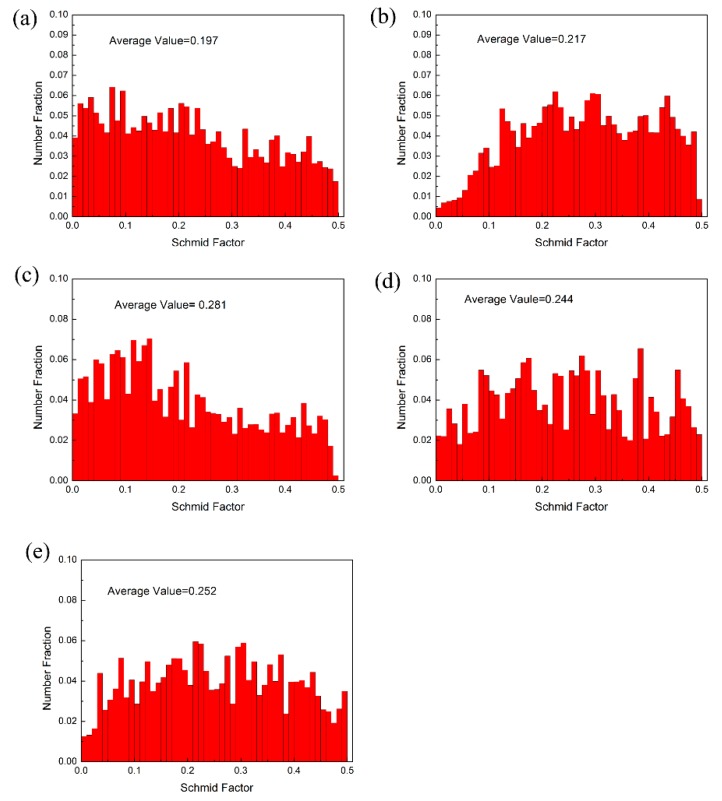
Schmid factor distributions of (**a**) as-received, (**b**) PCA200, (**c**) PCA 300, (**d**) PCA 400, and (**e**) PCA 500.

Schmid Factor distributions (SF) for the basal <a> slip system of various annealed AZ31 Mg alloy sheets are shown in Figure 6. It is obvious that the as-received sheet exhibits a typical basal texture and the SF of the basal plane is rather small. In other words, basal slips cannot be easily activated during the deformation, which results in a poor ductility. However, the rotated orientation of grains is a soft orientation for basal slips after precompression and annealing. The average Schmid Factor values consistently increased with the annealing temperature increasing (0.197 to 0.217, 0.244, 0.252, and 0.281 for PC200, PC300, PC400, and PC500, respectively). A higher basal Schmid factor has a significant contribution in accommodating the deformation, implying that the texture is much more favorable for <a> basal dislocation slips during the subsequent deformation, and thus improves the formability of Mg alloy sheets.

### 3.2. Mechanical Properties

**Figure 7:**
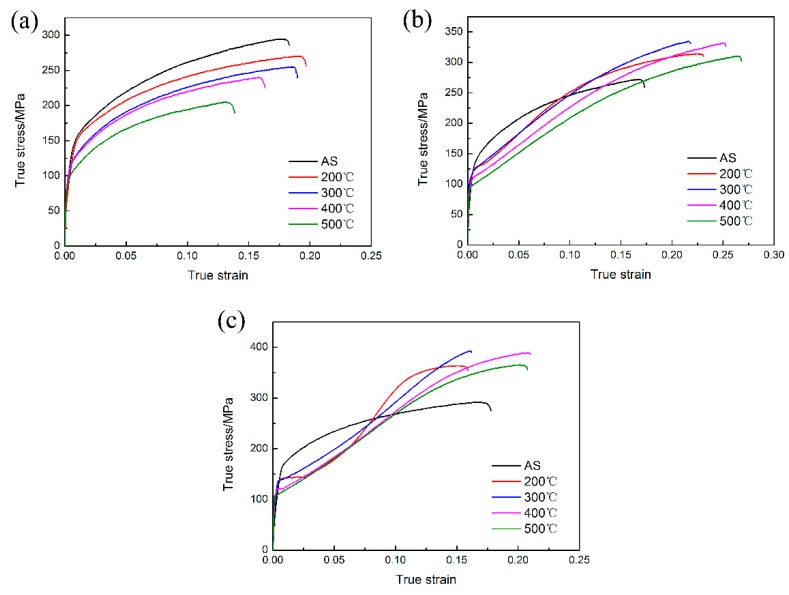
True stress vs strain curves PCA sheets along different directions: (**a**) RD, (**b**) 45°, and (**c**) TD.

Figure 7 shows the true stress vs strain curves of the investigated specimens along three directions, namely RD, 45, and TD. The mechanical characteristics, including 0.2% yield strength (YS), the ultimate strength (UTS), failure elongation (FE), strain hardening coefficient (n-value), and the Lankford value (r-value) are also summarized in Table 1. It can be seen that the flow stress decreases as the annealing temperature increases when there is tension along RD, resulting in concurrent drops in YS and UTS. As mentioned in the previous section, the grain size increased gradually with the annealing time and temperature increasing. According to the Hall–Petch relationship (σ_s_ = σ_0_ + kd^−1/2^), the smaller the grain size, the higher the yield strength will be [18]. Besides, TD-inclined texture was kept in precompressed AZ31 Mg sheets even after annealing process; this titled orientation with a higher SF (as shown in Figure 6) which results in a decrease of YS. Thus, the reduced yield strength was obtained in PCA sheets. Similarly, YS exhibits a decreasing trend along 45° and TD. In case of tension along TD, interestingly, a concave platform occurred in PCA sheets. Hama et al. [19] reported that the detwinning started when a reverse load was applied on the pre-twinned Mg alloys, and as a result a concave platform emerged in the tensile curves. However, all of the {10–12} tensile twins disappeared after recrystallization in this study. Thus, this phenomenon may be related to the rerotation of grains since the twinning orientation is inherited by the recrystallized grains. The fracture elongation (FE) also decreases after the annealing process, which is owing to the grain growth induced by annealing. The highest and lowest yield strength is obtained along TD and RD, respectively. The YS differences are measured about 25 MPa in sample A, however, it decreases to 8.6, 7.8, 3.3, and 6 MPa for PC200, PC300, PC400, and PC500, respectively. This is mainly owing to the presence of a strong basal texture in the as-received Mg alloy sheet, which results in a strong yield anisotropy. However, after the rotation of basal planes toward TD, yield anisotropy was reduced remarkably.

Table 1 also shows the strain hardening coefficient (n-value) and Lankford coefficient (r-value) of various PCA samples. From Table 1, it can be seen that average n-value is slightly reduced after the precompression and annealing process, implying that the process has no meaningful effect on n-value. A higher n-value causes an increase in hardenability of Mg alloys at room temperature. Table 1 shows that the Lankford coefficient (r-value) decreased gradually with the annealing temperature increasing. As is well-known, the width strain is always governed by prismatic <a> slip, while the thickness strain is accommodated by a pyramidal <c + a> slip and twinning during plane deformation of Mg alloy sheets. In as-rolled Mg alloy sheets with a strong basal texture, the thickness deformation cannot be generated which results in a larger r-value. However, in PCA Mg alloy sheets with a TD-inclined texture, the orientation of rotated grains is much more favorable for basal < a > slips, and thus, major deformation along the thickness can be accommodated by basal slips, which results in a smaller r-value. Besides, {10–12} tensile twins may activate in coarse grains. Fan et al. [20] proved that the propensity of deformation twinning reduced as grain size decreased. This may be responsible for the smallest average r-value on PC Mg alloy samples annealed at 400 °C.

**Figure 8:**
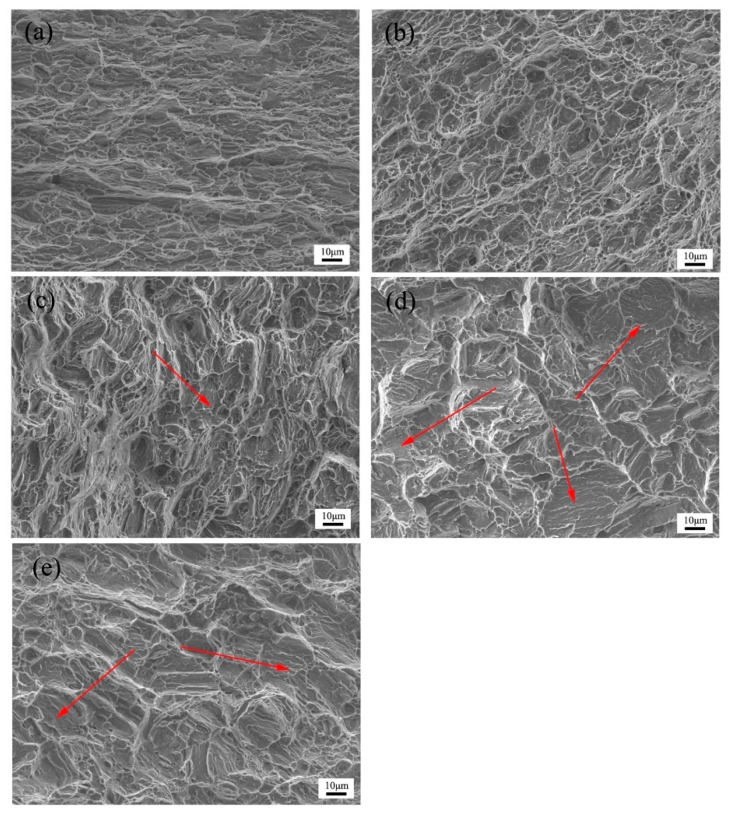
Fracture surfaces of (**a**) as-received, (**b**) PCA200, (**c**) PCA300, (**d**) PCA400, and (**e**) PCA500 Mg alloy samples.

A collection of features observed on fracture surfaces of the tensile broken specimens is shown in Figure 8. As seen in Figure 8a, the fracture surface of sample A exhibits a quasi-cleavage fracture with a small amount of local dimples, representing poor plasticity. However, in the case of PCA200 samples, a large number of dimples and some tearing edges were observed on the fracture surfaces, which indicates a markedly ductile fracture. This is related to the residual twins after annealing whose orientation had a softening effect on the subsequent deformation. However, as shown by arrows in Figure 8c–e, with increasing the annealing temperature, the fraction of cleavage planes increased, especially on PCA400 specimens which indicate a typical brittle rupture. As previously mentioned in Figure 2, the largest grain size (32.9 μm) was obtained in the samples annealed at 400 °C, confirming the occurrence of the brittle fracture mode.

### 3.3. Formability

**Figure 9:**
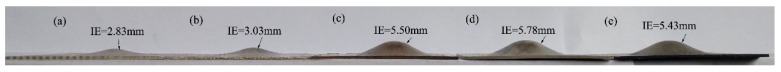
Erishen values (IE) of: (**a**) as-received, (**b**) PCA200, (**c**) PCA300, (**d**) PCA400, and (**e**) PCA500.

Erishen test specimens are shown in Figure 9. It can be seen that IE-value of the as-received sample is only 2.83 mm which increases by 3.03, 5.50, 5.78, and 5.43 mm on PC200, PC300, PC400, and PC500 samples, respectively. The results show that the stretch formability is remarkably improved by 104.2% in PCA400 Mg alloy samples. However, the biggest grain size is also obtained in this specimen which is in contrary to the Hall–Petch relationship. It is worth noting that the basal plane rotation from ND to TD leads to a larger SF, and thereby, a higher activity of basal slip can be used to accommodate the thickness deformation, which eventually results in the improvement of stretch formability. Huang et al. [21] reported the resistance of sheet thinning will be enhanced with a smaller r-value. The IE-value continuously increased with increasing annealing temperature, giving the largest value of 5.78 mm for PCA400 samples which then dropped to 5.43 mm for the PCA500 sample. This is ascribed to the reduced anisotropy and activation of {10–12} tensile twinning in coarse grains during the stretch deformation, which is in a good agreement with the smallest YS differences obtained in PCA400.

**Figure 10:**
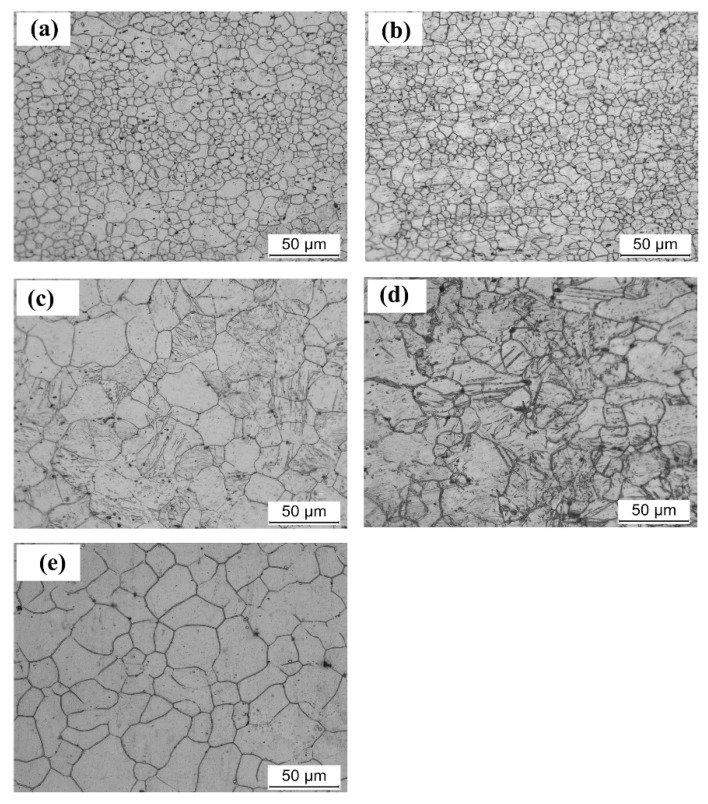
Optical microstructures on the top of the Erishen tested samples (**a**) as-recieved, (**b**) PCA200, (**c**) PCA300, (**d**) PCA400, and (**e**) PCA500.

In order to evaluate the role of twins, the microstructures on the top of the Erishen tested specimens are measured. Figure 10 shows the microstructures which are close to the fracture region of the Erishen tested specimens. It can be seen that the microstructure is similar to that of their as-received counterpart. However, for PCA200 samples, initial twinning lamellas are disappeared. This might be attributed to the detwinning behavior. Since detwinning happens when an inverse load is applied to pre-twinned Mg alloys, in concurrence with results reported by Wu et al. [22]. Huo et al. [23] indicated the inside region of Mg alloy sheet was under compressive strain, while the outer region underwent tension during the stretch deformation. Accordingly, the inverse tensile load might be responsible for the occurrence of detwinning. However, in PCA300 and PCA400 Mg alloy sheets, twins emerged in the microstructures after the Erishen test. Besides, the largest twining volume fraction was obtained in PCA400 specimens which featured the largest grain size. As noted before, Song et al. [24] reported that twining played an important role in accommodating the thickness deformation which, in turn, helps to improve the stretch formability in Mg alloy sheets. However, due to the increased intensity of rotated basal planes, the grain orientation is less favorable for {10–12} tensile twins. Thus, it can be deduced that combined effects of texture modification (rotation of basal planes) and twinning plays an important role in improvement of the formability of Mg alloy sheets. 

## 4. Conclusions

In this study, precompression along TD followed by an annealing treatment at different temperatures was conducted with the aim to induce grain growth in AZ31 Mg alloy thin sheets. It was found that with increasing the annealing temperature from 200 to 500 °C alongside the occurrence of static recrystallization grain size gradually increased due to the strain-induced boundary migration (SIBM). The biggest grain size was obtained when annealing at 400 °C. After short time annealing, it can be seen that there is little change on microstructure in as-received alloys, however, the recrystallization almost completes in 120 s growth ceases. Thus, the energy for recrystallization is mainly precompression strain not the thermal activation effect at higher temperature. Due to the consuming of {10–12} tensile twins by the new nucleated grains, the orientation of twins are inherited and the orientation of the recrystallized grains was close to TD. Due to the grain growth, both the yield strength and ductility of precompression and annealed sheets decreased, while the stretch formability improved. The IE-value increased from 2.83 mm for as-received Mg sheets to 3.03, 5.50, 5.78, and 5.43 mm after precompression and annealing at 200, 300, 400, and 500 °C, respectively, giving a remarkable improvement of approximately 104%. This outstanding enhancement was found to be mainly related to the rotated grain orientation and the activation of {10–12} tensile twinning during stretch deformation. 

## Figures and Tables

**Table 1:** Mechanical properties of various temperatures annealed AZ31 magnesium alloy sheets.

Annealing Condition		AS	200 °C	300 °C	400 °C	500 °C
YS/MPa	RD	144.5	134.1	129.1	117.7	102.9
	45°	146.7	128.8	118.6	112.2	105.8
	TD	169.5	142.7	136.9	121.3	108.6
UTS/MPa	RD	304.3	290.2	255.1	239.3	204.5
	45°	283.9	314.3	324.7	332.1	300.7
	TD	291.6	363.8	390.5	379.5	361.5
FE/%	RD	17.3	18.5	17.5	14.9	12.5
	45°	17.9	22.9	21.9	25.2	26.8
	TD	17.8	15.9	16.2	21.1	19.8
n	RD	0.437	0.438	0.408	0.389	0.396
	45°	0.425	0.426	0.395	0.437	0.411
	TD	0.388	0.321	0.391	0.399	0.469
$\bar{n}$		0.418	0.403	0.397	0.415	0.421
r	RD	2.361	0.532	0.621	0.371	0.421
	45°	3.142	1.482	1.121	1.101	1.272
	TD	1.532	0.171	0.472	0.552	0.798
$\bar{r}$		2.544	0.916	0.833	0.781	0.941
